# Mesenteric Ischemia due to Thrombosis Involving the Aorta, Celiac Artery, and Superior Mesenteric Artery in a Young Female with Protein C Deficiency

**DOI:** 10.7759/cureus.6151

**Published:** 2019-11-14

**Authors:** Muhammad Hamza, Huma Sabir Khan, Amna Arshad, Muhammad Ahmed, Muhammad Hanif

**Affiliations:** 1 Surgery Unit Ii, Benazir Bhutto Hospital, Rawalpindi Medical University, Rawalpindi, PAK; 2 Surgical Unit II, Benazir Bhutto Hospital, Rawalpindi Medical University, Rawalpindi, PAK; 3 Surgical Unit Il, Benazir Bhutto Hospital, Rawalpindi Medical University, Rawalpindi, PAK

**Keywords:** mesenteric ischemia, thrombus, fibroid, protein c deficiency

## Abstract

An arterial thrombus affecting the descending aorta, celiac artery, and superior mesenteric artery at the same time, resulting in mesenteric ischemia and splenic infarction, is a very rare phenomenon. We report a case of a 35-year-old, unmarried female, gravida 0 para 0, who presented with abdominal pain, vomiting, and constipation for two days. Computed tomography (CT) scan showed thrombi in the descending aorta to the celiac axis and superior mesenteric artery with splenic infarction, bowel ischemia, hepatic portal venous air, and uterine fibroid. The diagnosis of arterial thrombotic mesenteric ischemia was made. An exploratory laparotomy was performed. Gangrenous intestine resection was done with ileojejunostomy and feeding ileostomy.

## Introduction

Acute mesenteric ischemia (AMI) is a surgical emergency that requires prompt diagnosis and early treatment, otherwise; it can have a catastrophic effect on patient survival. This disease has a very high morbidity and mortality rate (80% to 100%) [1-2]. AMI can be subdivided into four types: acute arterial thrombotic mesenteric ischemia, acute arterial embolic mesenteric, acute venous mesenteric ischemia, and non-occlusive mesenteric ischemia. Arterial thrombotic mesenteric ischemia accounts for 30% of all ischemic events. It mostly involves the proximal part of the superior mesenteric artery [1,3]. Clinical symptoms and signs are almost always non-specific, and the patient mostly lands as a case of acute abdomen in the emergency setting [1-3]. A better understanding of this disease can help clinicians for the early diagnosis and treatment of this disease.

We herein report a young female in Rawalpindi, Pakistan with arterial occlusive mesenteric ischemia due to thrombus involving the descending aorta, celiac artery, and superior mesenteric artery, presenting as a case of acute abdomen. This is a rare case as extensive thrombus extending from the descending aorta to the celiac artery and superior mesenteric artery has not been previously reported.

## Case presentation

A 35-year-old Pakistani non-smoker, unmarried female, gravida 0 para 0, presented to the emergency with a complaint of sudden, severe, and generalized abdominal pain for two days. The pain was non-radiating and persistent and was associated with three episodes of vomiting containing food particles and absolute constipation. There was no history of fever, upper/lower gastrointestinal bleeds, intermittent claudication, rest leg pain, diarrhea, and rectal bleeds/blood in stools or trauma. There was no record of cardiac, mental, respiratory, urinary, or genital diseases. She did not have any record of any prior illness, surgery, or allergy. There was no significant family or psychosocial history. Her menarche commenced at the age of 13 years with later regular cycles.

She presented to the emergency with tachycardia, tachypnea, pallor, and dehydration. The abdomen was distended, tensed, and bowel sound was absent. There was a firm hard mass palpable in the lower abdomen extending from the pelvis to the umbilicus. The lower limit of the mass was not reachable. The fluid thrill was absent. The abdominal bruit was absent. On the digital rectal examination, the rectum was empty, but the finger was not bloodstained. The vaginal and genital examination was normal.

**Figure 1:**
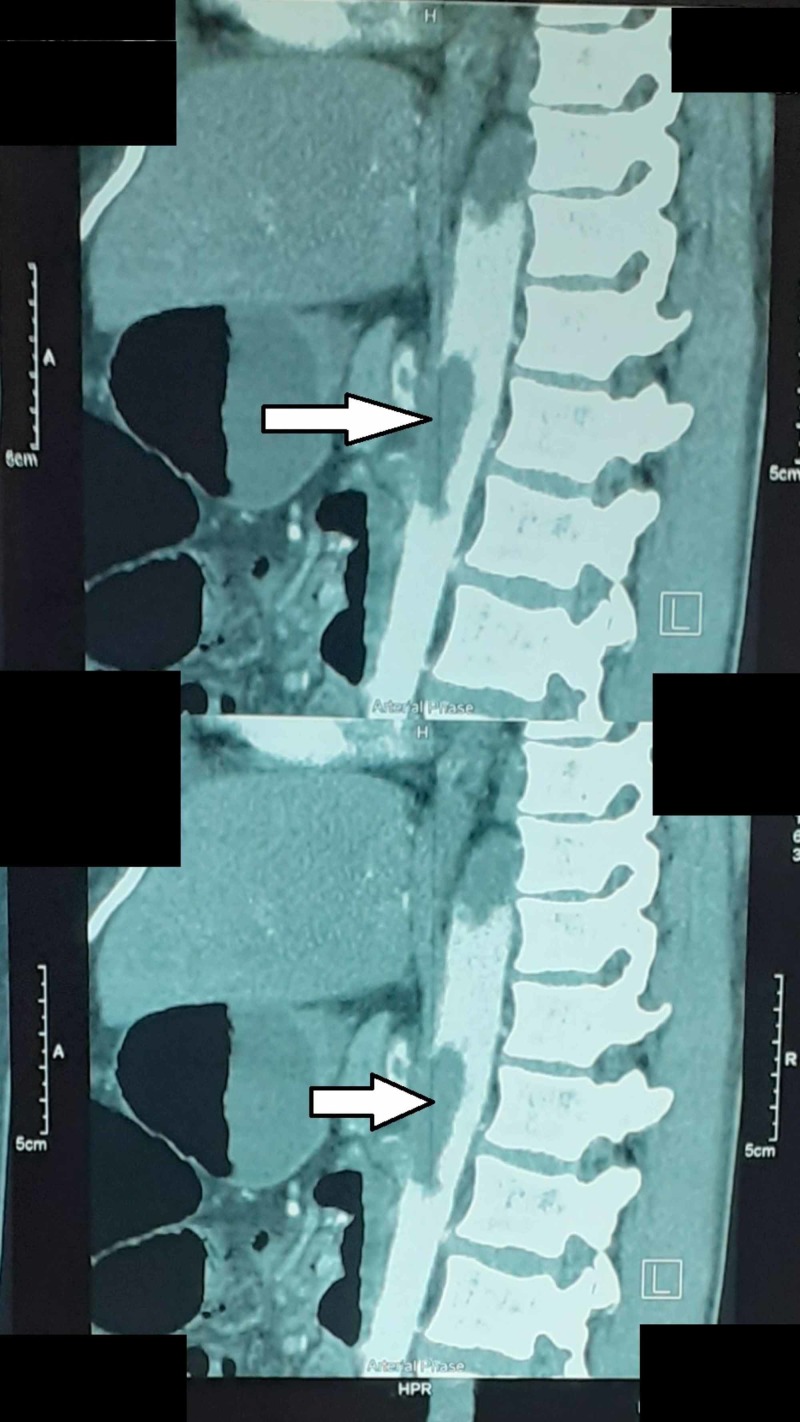
CTA showed aortic thrombus extending into the celiac axis and superior mesenteric artery CTA, computed tomography angiography

The investigation revealed that hemoglobin was 7.7 g/dl, total leukocyte count was 15.8 x 109 /UL, and platelet count was 577,000 /UL. Prothrombin time, activated partial thromboplastin time, liver function test, renal function test, and serum electrolyte and blood sugar levels were normal. Hepatitis B and C profiles were negative. The pregnancy test was negative. Chest X-rays, electrocardiogram (ECG), and echocardiography were normal. Erect abdominal X-rays showed signs of bowel obstruction. Abdominal ultrasonography (USG) and CT scan of the abdomen/pelvis showed multiple uterine fibroids, dilated gut loops, splenic infarction, bowel ischemia, and hepatic portal venous air. Computed tomography angiography (CTA) showed two thrombi in the descending aorta. One aortic thrombus was extending from the thoracic vertebrae nine to 12 along the right lateral wall of the aorta. The other aortic thrombus was extending from the thoracic vertebra 12 to the celiac axis and superior mesenteric artery (Figure 1).

The diagnosis of AMI due to arterial thrombus was made, and an exploratory laparotomy was planned. The patient was counseled and informed consent was taken. Intraoperative findings were 150 mL pus in the abdomen, and the small intestine (one foot from the duodenojejunal flexure to four feet from the ileocecal junction) was gangrenous. There were dense adhesions and fibroid uterus. Resection of the gangrenous bowel was done. Ileojejunostomy and feeding ileostomy were made. There were no intraoperative complications. The patient remained admitted for two weeks. Subcutaneous injection of enoxaparin was started initially; then the patient was shifted to tablet rivaroxaban 10 mg OD and tablet aspirin 75 mg OD as per hematologist consultation. During this period, the feeding ileostomy was displaced, and so a distal loop of ileojejunostomy, i.e. ileum, was used for feeding purpose. The mutation for JAK-2 kinase was negative.

**Figure 2:**
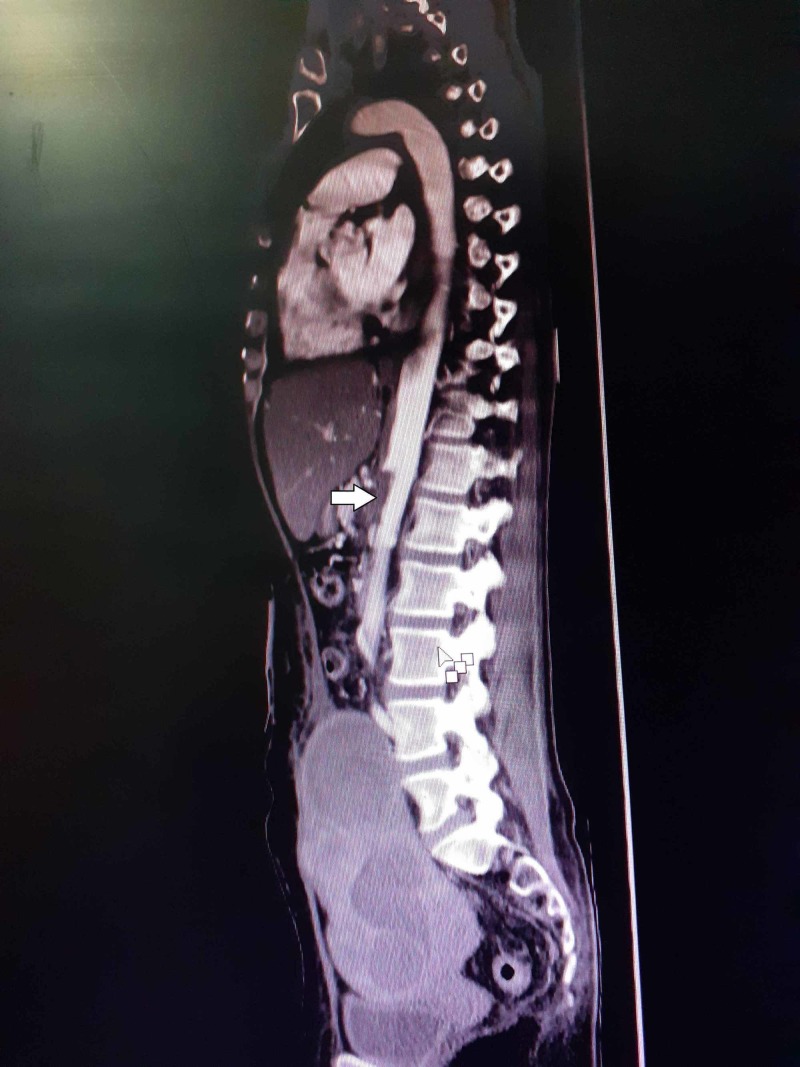
CTA showed non-dissolution of aortic thrombus at three months follow-up CTA, computed tomography angiography

The patient presented to us after one month. Her Doppler study for intestinal blood flow suggested normal flow. After three months, her CTA showed non-dissolution of aortic thrombus (Figure 2).

The reversal of ileojejunostomy was postponed. The patient had a favorable follow-up during these three months. Long-term anticoagulation was advised for the thrombus dissolution.

## Discussion

The importance of AMI cannot be undermined as the understanding of this disease is crucial for all physicians and surgeons around the globe. This is truly a surgical emergency that requires prompt diagnosis and effective early management to enhance patient survival. The delay in diagnosis can be very catastrophic [3]. AMI is a rare phenomenon, especially considering this disease in a young female. Thromboembolic phenomena are the most common cause of mesenteric ischemia, which may be associated with several prothrombotic disorders [4].

Protein C is an enzyme that inactivates the coagulation factor V, Leiden and VIII. Protein S increases the action of protein C. Protein C and S deficiencies cause a prothrombotic state [4]. Patel et al. have reported ischemic stroke in a 25-year-old male having both protein C and S deficiencies [5]. Isolated protein S deficiency has been reported in the literature as a cause of mesenteric ischemia [4]. There is no case report of isolated protein C deficiency associated with this disease. Maqbool et al. have reported pulmonary embolism and myocardial infarction in a 37-year-old male having isolated protein C deficiency [6]. Aortic thrombosis linked to isolated protein C deficiency is rare [7]. Moreover, extensive thrombus extending from the descending aorta to the celiac artery and superior mesenteric artery causing symptomatic mesenteric ischemia and splenic infarction has not been reported earlier as per our knowledge.

The patient having AMI presented with non-specific symptoms and signs. The key to diagnosis is a high index of clinical suspicions. Severe abdominal pain that does not match with the examination is classic for this disease. According to the guidelines of the World Society of Emergency Surgery, CTA (diagnostic sensitivity of 96% and a specificity of 94%) should be performed as early as possible when there is a suspicion of this disease. Since this disease carries a very high mortality risk and has a very poor prognosis, resuscitation, antibiotics, and IV heparin (if not contraindicated) should be immediately started. The majority of treatment options depend on the following factors: presence/absence of signs of peritonitis, presence/absence of ischemia but viable intestine, the general condition of the patient, and etiology of mesenteric ischemia. If signs of peritonitis are observed, then laparotomy should be performed [3]. The aim of laparotomy is the assessment of the viability of the bowel, revascularization, and the resection of the necrotic bowel. In our patient, the resection of the gangrenous gut was done. Ileojejunostomy and feeding ileostomy were made. During this period, the feeding ileostomy tube was displaced. It was decided that the distal loop of ileojejunostomy, i.e. ileum, can be used for feeding purposes. The patient tolerance was good. We did not perform second-look laparotomy as we found it unnecessary. Revascularization during a laparotomy was not done because of high surgical risk, late presentation of the patient, and extensive thrombosis of the blood vessels causing significant bowel ischemia. The patient was put on long-term anticoagulation therapy for the dissolution of thrombi. The patient had a favorable follow-up for three months.

## Conclusions

AMI can be triggered by many prothrombotic factors. Protein C deficiency can also lead to this catastrophic disease. Thus, AMI is an emergency that requires immediate medical and surgical management with supportive intensive care for patient survival.

## References

[1] Mastoraki A, Mastoraki S, Tziava E (2016). Mesenteric ischemia: pathogenesis and challenging diagnostic and therapeutic modalities. World J Gastrointest Pathophysiol.

[2] Chang RW, Chang JB, Longo WE (2006). Update in management of mesenteric ischemia. World J Gastroenterol.

[3] Mazzei MA (2018). Acute mesenteric ischemia: Guidelines of the World Society of Emergency Surgery: a brief radiological commentary. World J Emerg Surg.

[4] Romano N, Prosperi V, Basili G (2011). Acute thrombosis of the superior mesenteric artery in a 39-year-old woman with protein-S deficiency: a case report. J Med Case Rep.

[5] Patel ML, Sachan R, Seth G (2013). Combined deficiency of proteins C and S: ischaemic stroke in young individuals. BMJ Case Rep.

[6] Maqbool S, Rastogi V, Seth A, Singh S, Kumar V, Mustaqueem A (2013). Protein-C deficiency presenting as pulmonary embolism and myocardial infarction in the same patient. Thromb J.

[7] Ueda K, Morishita E, Shiraki H, Matsuoka S, Imashuku S (2019). Aortic mural thrombus associated with congenital protein C deficiency in an elderly patient. J Atheroscler Thromb.

